# The Emerging Role for RNA Polymerase II in Regulating Virulence Gene Expression in Malaria Parasites

**DOI:** 10.1371/journal.ppat.1004926

**Published:** 2015-07-16

**Authors:** Uchechi E. Ukaegbu, Kirk W. Deitsch

**Affiliations:** Department of Microbiology and Immunology, Weill Medical College of Cornell University, New York, New York, United States of America; University of Wisconsin Medical School, UNITED STATES

## Introduction

*Plasmodium falciparum* causes the most severe form of human malaria and is responsible for a significant public health burden in the developing world. These protozoan parasites invade circulating red blood cells (RBCs) and maintain prolonged infections through an intricate gene-expression switching mechanism that enables immune evasion through antigenic variation [1]. One of the families of genes responsible for this evasion is called *var*; it is made up of ~60 members, of which approximately two-thirds are found located within subtelomeric heterochromatic regions of the *P*. *falciparum* genome, with the remaining third of the family arranged in clusters located in similarly heterochromatinized areas within the internal regions of the chromosomes. The *var* gene family encodes *P*. *falciparum* erythrocyte membrane protein 1 (PfEMP1), a protein displayed on the surface of infected RBCs and considered the primary antigenic determinant required for cytoadherence and sequestration of infected cells, thus enabling them to avoid circulation through the spleen. Only one *var* gene is expressed at a time, while the other 59 remain transcriptionally silent, and which gene is active switches over the course of an infection. This process allows the parasites to maintain chronic infections through an ever-changing display of PfEMP1 antigens to the immune system. Structurally, each *var* gene consists of two exons flanking a single, conserved intron with a bidirectional promoter that transcribes noncoding RNAs [2–4].

Although an understanding of the mechanisms that lead to coordinated switching within the *var* gene family have remained elusive, it has become clear in recent years that epigenetic components, particularly histone modifications, play major roles in determining whether an individual gene will be active or silent. Many of the histone modifications involved in antigenic variation, as well as other aspects of parasite development, have been catalogued. For example, trimethylation of histone H3 at lysine 4 (H3K4me3) denotes transcriptionally active genes [5], including the single active *var* gene. In contrast, H3K9me3 marks the 59 silent *var* genes [6,7]. Interestingly, H3K9me3 and another mark, H3K36me3, are very limited in their distribution throughout the genome and are found primarily at gene families that encode variant antigens like *var* [5,8]. How the enzymes that deposit these marks are recruited to very limited regions of the genome is poorly understood. This short review aims to expand on recent work that sheds light on the recruitment of histone modifiers to narrow regions of chromosomes, specifically *var* genes, by way of the C-terminal domain of RNA polymerase II (RNA pol II CTD).

## The C-Terminal Domain (CTD) of RNA Pol II: A Platform for Coordinating Transcription

**Fig 1:**
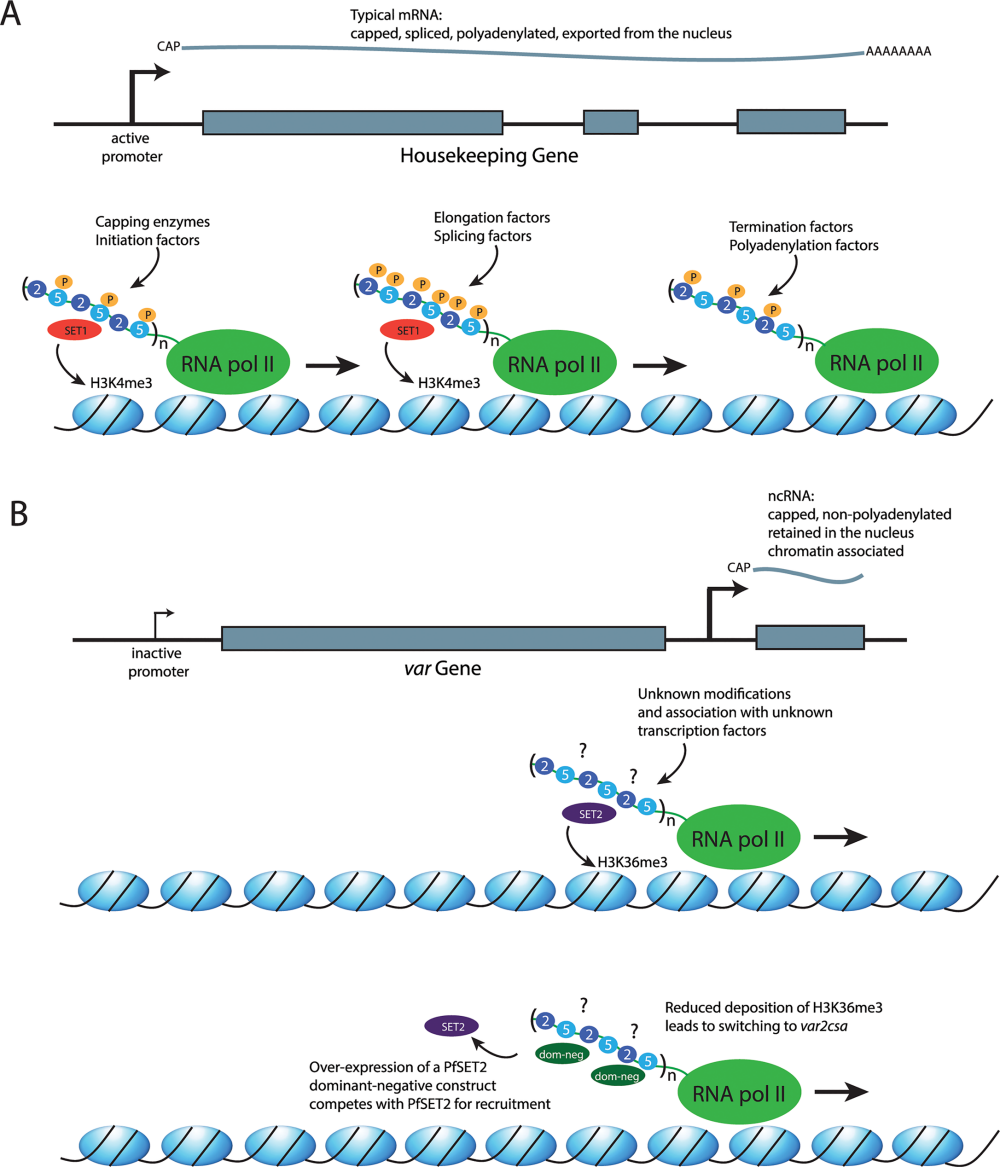
The role of the RNA polymerase II CTD in recruiting different factors to the transcription complex. (A) Transcription of a typical mRNA. Top: a representation of a housekeeping gene with three exons (gray boxes) and two introns. Recruitment of an mRNA producing RNA pol II complex to the promoter results in the production of a transcript that is capped, spliced, polyadenylated, and exported from the nucleus for translation. Bottom: a representation of the DNA strand wrapped around histones (blue ovals). The RNA pol II complex is shown in green, with the CTD shown as an extension. In eukaryotes, the CTD consists of a series of repeats of the amino acid sequence YSPTSPS/K. The serines in the second and fifth positions of each repeat are shown as blue circles labeled 2 and 5, respectively. Three repeats are shown, although the number can vary according to species (n). In *P*. *falciparum*, 12–17 repeats have been reported. Phosphorylation of the serines are shown as orange circles labeled with a P and are found primarily on serines in the fifth position near the beginning of transcription, on the serines in both positions at the middle of the transcription unit, and at the serines in the second position where transcription terminates. The alternate phosphorylated states mediate recruitment of different factors required for mRNA production and maturation, as well as histone modifiers, including SET1 (red oval), which deposits the epigenetic mark H3K4me3 concurrent with the production of an mRNA. Thus, this mark is found at transcriptionally active regions of the genome. (B) A model for transcription of a noncoding RNA (ncRNA) at *var* loci. Top: a promoter found within the introns of *var* genes leads to the production of ncRNAs that are capped but not polyadenylated. In addition, they are retained within the nucleus and associated with chromatin. Middle: the RNA pol II complex is engaged in transcribing a noncoding RNA; however, the modifications of the serines at positions 2 and 5 of the CTD and the transcription-related factors that are recruited to the complex are unknown (?). PfSET2 (purple oval) has been shown to bind directly to the CTD and to deposit the epigenetic mark H3K36me3 at both active and silent *var* genes. This is consistent with its recruitment by RNA pol II when it is transcribing the ncRNA from the promoter located within *var* introns. Bottom: a dominant-negative version of PfSET2 (dark green ovals) that is not capable of depositing the H3K36me3 mark can compete with the endogenous wild-type PfSET2 for binding to the CTD. Overexpression of the dominant-negative protein reduces the efficiency of PfSET2 recruitment to *var* loci and leads to switching to *var2csa*.

As with all eukaryotes, malaria parasites possess three different RNA polymerases. RNA pol I transcribes the ribosomal RNAs (with the exception of 5S), while pol III transcribes the 5S ribosomal RNA (rRNA), transfer RNAs (tRNAs), and several small RNAs, including some small nucleolar RNAs (snRNAs). RNA pol II mediates expression of protein-coding genes by transcribing the corresponding messenger RNAs (mRNAs). Central to coordinating the various steps in mRNA production is the systematic post-translational modification, specifically phosphorylation, of the CTD of Rpb1, the largest subunit of RNA pol II (Fig 1A) [9]. This enables the stepwise recruitment of various factors involved in the regulation of the transcription cycle as well as RNA processing, RNA export, and chromatin remodeling. In most eukaryotes, the RNA pol II CTD has a flexible structure comprised of a highly conserved tandem array of Tyr1-Ser2-Pro3-Thr4-Ser5-Pro6-Ser/Lys7 heptad repeats. As RNA pol II moves through the transcription of a gene, the repeats undergo sequential changes in the phosphorylation status of Ser2 and Ser5 whereby phosphorylation of Ser5 alone is associated with transcriptional initiation, concurrent phosphorylation of both Ser2 and Ser5 when the polymerase is engaged in elongation, and phosphorylation of Ser2 alone at termination. The transcription cycle is not the only process that is dependent on appropriate phosphorylation of the serine residues. Nuclear factors such as capping enzymes and histone modifiers, as well as splicing factors, are equally dependent on the phosphorylation status of the CTD for appropriate recruitment to the transcription complex [10]. For example, the histone methyltransferase SET1 binds to the CTD when it is phosphorylated at serine 5, thus marking chromatin at regions of the genome that have recently been transcribed (Fig 1A) [11].

## The Evolution of the RNA Pol II CTD in Malaria Parasites

Comparison of RNA pol II CTD sequences from different evolutionary lineages shows that CTD length is loosely correlated with genome size [12]. Moreover, individual lineages tend to have tightly conserved heptad numbers; for example, all mammals possess 52 repeats in their CTDs. Interestingly, genome sequences of several protozoan lineages reveal some peculiar and intriguing differences in their CTDs. Trypanosomes and species of *Trichomonas* display traces of the standard CTD heptads but not the canonical repeat structure [12–14]. *Plasmodium*, on the other hand, has a bona fide RNA pol II CTD; however, members of this genus display an unprecedented plasticity in the length and composition of the CTD, something not observed in any other eukaryotic lineage (described in detail in [15]). Malaria parasites that infect rodents, birds, reptiles, and bats all possess a remarkably short CTD consisting of eight conserved heptad repeats. In contrast, the primate parasites *P*. *knowlesi*, *P*. *vivax*, *P*. *falciparum*, and *P*. *cynomolgi* show an expanded number of heptads that is highly variable, both within and between species, ranging from 12 to 17 repeats. More precisely, primate parasites possess an expansion of a specific heptad near the middle of the CTD that contains a lysine residue in the seventh position. The function of this heptad expansion is unknown, although it has been noted that the increased heptad number is correlated with the use of epigenetic memory for antigenic variation [16]. This observation is even more intriguing considering recent studies implicating the CTD in the recruitment of histone modifiers to variant antigen encoding genes, in particular *var* genes (see below).

## The Role of the RNA Pol II CTD in the Recruitment of Histone Modifiers in *P*. *falciparum*

Similar to its counterparts in higher eukaryotes, recent work indicates that the RNA pol II CTD of malaria parasites serves as a platform for the recruitment of nuclear factors for gene regulation and cross talk between chromatin and RNA pol II [16]. The first nuclear factor for which recruitment by the CTD has been demonstrated in *P*. *falciparum* is PfSET2, a histone methyltransferase that deposits a trimethylation mark on H3K36. The H3K36me3 mark is tightly coupled to transcription by RNA pol II in both yeast and mammalian cells; therefore, it is found throughout transcribed regions of the genome of these organisms [17,18]. However, in the *Plasmodium* genus, orthologues of SET2 are found only within the genomes of parasites that infect primates, thus mirroring the RNA pol II CTD expansion [16]. Further, the H3K36me3 mark is not found distributed throughout the genome but rather is only present within the telomeric repeats and the large, multicopy gene families involved in antigenic variation—for example, *var* genes [8]. This implies that malaria parasites have evolved a way to recruit PfSET2 only to these narrow regions of the genome. In higher eukaryotes, SET2 is recruited to all transcribed regions of the genome by binding directly to the CTD of RNA pol II while it is actively engaged in transcription, specifically when the serine residues in positions 2 and 5 of the heptad repeats are phosphorylated [18,19]. Interestingly, in vitro coimmunoprecipitation assays show specific binding of PfSET2 to the CTD when both Ser2 and Ser5 are unphosphorylated, and phosphorylation of the CTD disrupted PfSET2 binding, indicating significant differences in how PfSET2 is recruited by RNA pol II in *Plasmodium* when compared to higher eukaryotes [16].

## The Role of RNA Pol II CTD Recruitment of PfSET2 in Antigenic Variation in *P*. *falciparum*

Studies employing a dominant-negative version of PfSET2 provided evidence for a direct role for the RNA pol II CTD in regulating *var* gene expression and antigenic variation [16]. As described above, only a single member of the *var* gene family is transcribed at a time, with switching of the expressed copy resulting in antigenic variation. A truncated version of PfSET2 consisting solely of the CTD binding domain competes with the endogenous PfSET2 for binding to the CTD, thus functioning as a “dominant-negative” when overexpressed in transgenic parasites (Fig 1B). This results in profound changes in *var* gene expression, inducing rapid switching to a single *var* gene called *var2csa* and indicating that recruitment of PfSET2 through CTD binding plays a key role in *var* gene regulation. Why this particular *var* gene becomes activated when PfSET2 recruitment by RNA pol II is disrupted is not clear. Several studies have documented that *var* gene switching is not random but rather follows a hierarchy of switching frequencies and that expression switching within the *var* repertoire is somehow coordinated within a network [20–25]. It is tempting to speculate that the selective activation of *var2csa* in these experiments might provide clues on the molecular basis of this proposed regulatory network; however, the details remain unknown.

The H3K36me3 mark deposited by PfSET2 is found within the chromatin of all *var* genes, both active and silent, indicating that PfSET2 is recruited regardless of whether an mRNA is being transcribed [8,16]. If PfSET2 is recruited by binding to the CTD of RNA pol II, how does it get recruited to transcriptionally silent genes? In addition to the mRNA transcribed from the single active gene, RNA pol II also transcribes noncoding RNAs from all *var* genes, both active and silent [26]. The promoter responsible for transcribing the noncoding RNAs, located within a conserved intron found in each gene, was shown to be required for proper *var* gene regulation [27,28], consistent with a role in the recruitment of RNA pol II and PfSET2 to each *var* gene. The fact that RNA pol II is transcribing a noncoding RNA rather than an mRNA in this context might provide a mechanism for selective recruitment of PfSET2 to limited regions of the genome. The field of noncoding RNAs has exploded in recent years and encompasses many distinct molecular mechanisms (reviewed in [29]). Numerous noncoding RNAs of unknown function have now been identified in *P*. *falciparum*, indicating that similar mechanisms are likely to be at work in malaria parasites [30–33]. The CTD undergoes differential phosphorylation while transcribing mRNAs, and the phosphorylation patterns are likely different when noncoding RNAs are being transcribed. If so, the unique modifications could be required for binding of PfSET2, thus limiting recruitment to regions where ncRNAs are transcribed. This hypothesis is consistent with the in vitro observation that PfSET2 does not bind to a typically phosphorylated CTD [16]. The telomeric repeats, another region of the genome where PfSET2 is recruited, also transcribe noncoding RNAs [34,35], and the noncoding RNAs themselves could also play a role in chromatin assembly, as has been observed in higher eukaryotes [29]. These observations suggest a model in which RNA pol II can actively recruit different histone modifiers to different parts of the genome through alternative modifications to the CTD (Fig 1B). There is experimental evidence to support this model for *var* loci [16]; however, it could also apply to other regions of the genome that have been shown to transcribe noncoding RNAs, thus potentially serving as a more general mechanism for chromatin assembly and organization.

## Perspectives

In addition to PfSET2, there are eight additional SET-domain-containing proteins encoded in the *P*. *falciparum* genome [36] and another putative SET protein proposed to be localized to the single active *var* gene [37]. These proteins are presumed to be involved in regulating gene expression throughout the rest of the genome, as they are in higher eukaryotes. Additional work with RNA pol II will help determine what role it plays in mediating these processes. A more complete understanding of the uniqueness of the *Plasmodium* RNA pol II CTD and the peculiar nature of its phosphorylation code will undoubtedly cast considerable light on this subject, potentially unifying previous observations regarding specific deposition of histone modifications, noncoding RNAs, gene activation and silencing, and mutually exclusive expression.
